# Impact of mentalization, identity diffusion and psychopathology on nonsuicidal self‐injury among adolescents

**DOI:** 10.1002/jad.12422

**Published:** 2024-10-09

**Authors:** Ayşe Selma Yenen Menderes, Füsun Çuhadaroğlu

**Affiliations:** ^1^ Department of Child and Adolescent Psychiatry Hacettepe University Faculty of Medicine Ankara Türkiye; ^2^ Department of Child and Adolescent Psychiatry Ankara Atatürk Sanatory Education and Research Hospital Ankara Türkiye

**Keywords:** adolescent, identity diffusion, Mentalization, nonsuicidal self‐injury, psychopathology

## Abstract

**Introduction:**

Nonsuicidal self‐injury (NSSI) has become a major public health issue in adolescents. This cross‐sectional case‐controlled study aims to assess the impact of identity diffusion, psychopathology, and mentalization on NSSI in adolescence.

**Methods:**

The study sample consisted of 153 adolescents (76.5% girls; M_age_ = 15.6 years). The sample included 56 clinical cases of NSSI, 45 psychiatric cases without NSSI, and 52 healthy controls, all recruited in Ankara, Türkiye, from June 1, 2022, to August 31, 2022. Mentalization was assessed by “The Movie for the Assessment of Social Cognition” (MASC) which categorizes mentalizing as “correct mentalizing” “hypermentalizing” “undermentalizing” and “no‐mentalizing”. All participants filled in the Self‐Injurious Behavior Screening Questionnaire, Assessment of Identity Development in Adolescence (AIDA), and Youth Self Report (YSR), and Inventory of Statements About Self‐injury (ISAS).

**Results:**

The NSSI group showed lower mentalizing capacity than the healthy control group (*p* = .011), and more no‐mentalizing errors than the other two groups (*p* = .014). Identity diffusion scores were higher in the NSSI group than in the other two groups (*p* < .001). Multivariate logistic regression analysis showed that the presence of maternal psychiatric disorder (*p* = .019, *OR* = 5.21), identity diffusion (*p* = .007, *OR* = 1.02), no mentalizing (*p* = .049, *OR* = 1.28), and total psychopathology symptoms (*p* = .009, *OR* = 1.12) had a significant impact on NSSI.

**Conclusions:**

Current findings suggest that transdiagnostic approaches, including mentalization and identity diffusion, may contribute to a more comprehensive understanding of NSSI and to the development of clinical interventions.

## 1 INTRODUCTION

Nonsuicidal self‐injury (NSSI) is defined as direct and deliberate damage to one's own body tissue without suicidal intent (Klonsky et al., 2014). NSSI has become a major public health issue among adolescents all over the world (Lim et al., 2019). The lifetime prevalence of NSSI among adolescents has been reported to range from 8.0% to 26.3% in community samples worldwide (Swannell et al., 2014), with rates increasing up to 40% to 80% in clinical samples (Kerr et al., 2010). Typically, the first episodes of NSSI occur in early adolescence (ages 12‐14), with frequencies peaking in mid‐adolescence (ages 15‐16), followed by a decline during late adolescence and into adulthood (Gandhi et al., 2018; Plener et al., 2015). The prevalence of NSSI has been found to be higher among girls, particularly in early and clinical‐based studies (Muehlenkamp & Gutierrez, 2007; Ross & Heath, 2002; Xiao et al., 2022). However, some of the community‐based studies have found no difference between two genders (Jacobson & Gould, 2007; Swannell et al., 2014). Studies in both clinical and nonclinical adolescent samples have found increased rates of psychological problems among those with NSSI compared to those without NSSI (Brown & Plener, 2017; Lewis & Heath, 2015; Xiao et al., 2022). Adolescents with NSSI often have internalizing disorders with high rates of depression and anxiety symptoms (Glenn & Klonsky, 2013; Gratz et al., 2015). NSSI is often accompanied by other psychiatric disorders. Studies have reported that the most common comorbidities are depression, attention‐deficit/hyperactivity disorder, posttraumatic stress disorder, anxiety disorders, borderline personality disorder, conduct disorder, substance use disorders and dissociative disorders (Koposov et al., 2021; Ross & Heath, 2002; Turner et al., 2015).

### 1.1 Nonsuicidal Self‐Injury and identity diffusion

Identity development is a critical developmental task during adolescence. Successful identity development involves adolescents identifying and selecting goals, values, and ideals that hold personal significance, ultimately leading to identity synthesis (Erikson, 1968). Conversely, identity diffusion, which results from the failure of identity formation, leads to a loss of one's capacity for self‐definition and commitment to values, goals, or relationships, often accompanied by a painful sense of inconsistency (Kernberg et al., 2000). Identity diffusion, the lack of integration of the concept of self and significant others, is also a core element of borderline personality disorder (BPD) and leads to a wide range of maladaptive and dysfunctional behaviors (Benzi & Madeddu, 2017). Additionally, several studies have found that individuals who lack a stable identity experience more internalizing problems (Crocetti et al., 2011) and more disturbed eating behaviors, non‐suicidal self‐injury, and suicidal behaviors (Gandhi et al., 2017; Sokol & Eisenheim, 2016; Verschueren et al., 2018).

Increasing evidence suggests that identity disturbances are related to increased risk for NSSI (Claes et al., 2014; Gandhi et al., 2016; Kahveci & Cetin, 2020). In a population‐based study of high school students, NSSI was found to be negatively associated with identity synthesis and positively associated with identity confusion (Claes et al., 2014). These findings have been replicated in many cross‐sectional studies in both community and clinical samples (Claes et al., 2015; Gandhi et al., 2016; Luyckx et al., 2015a, 2015b). Identity disturbances have also been linked with the behavioral motivations or functions of NSSI (Gandi et al.,2016). Claes et al. (2015) reported that female patients with self‐injurious eating disorders, who had higher levels of identity confusion, engaged in NSSI to reduce the impact of undesirable or aversive emotional states. Breen et al. (2013) suggested that NSSI may serve as a means of developing a sense of self‐identity (“I am a self‐injurer”) through connection with others who engage in similar behaviors.

### 1.2 Nonsuicidal Self‐Injury and mentalization

Mentalization is defined as the ability to recognize that other people have different minds, to understand and mentally represent the mental states of oneself or others, such as intentions, beliefs and desires (Fonagy & Bateman, 2019). Thus, mentalization enhances empathy skills, social interaction and communication skills, increases perspective taking, and helps them establish close, trusting relationships (Fonagy & Allison, 2012). During adolescence, mentalization is crucial for managing developmental challenges, including rapid changes in social demands, such as adapting to more complex social environments and developing new close relationships with peers (Andrews et al., 2021; Desatnik et al., 2023). Theoretical models of NSSI have suggested that difficulties in interpersonal functioning (e.g., poor communication and social problem solving, maladaptive stress responses to social demands) increase the risk of developing and maintaining NSSI (Guerry & Prinstein, 2010; Nock, 2010).

Adolescents mostly engage in NSSI as a maladaptive coping strategy to relieve and reduce intense and distressing emotions (Klonsky, 2007). Existing research demonstrates relations between poor emotion regulation skills and NSSI (Klonsky, 2007; Nielsen et al., 2016; Wolff et al., 2019). The capacity to be aware of one's own and others’ mental states supports emotion regulation skills by promoting to recognize the mental states that underlie human behavior (Fonagy & Allison, 2012). Moreover, mentalization allows for psychic and symbolic representation of one's own inner state and is also thought to reduce NSSI behavior by enabling people to regulate and control their emotions (Rossouw et al., 2020). A randomized controlled trial of adolescents with NSSI found that mentalization‐based treatment (MBT‐A) was more effective than treatment as usual (TAU) in reducing self‐harm behavior (Rossouw & Fonagy, 2012).

To date, limited research has examined mentalization skills in studies of adolescents with NSSI, although some components of mentalization such as emotion recognition and empathy skills have been assessed. A study involving 20 female adolescent inpatients with NSSI using the Theory of Mind Assessment Scale (Th.o.m.a.s.) found that adolescents with NSSI had lower mentalization performance than healthy controls, and there was a negative correlation between mentalization performance and suicidality, NSSI frequency, and severity (Laghi et al., 2016).

### 1.3 The current study

Existing literature strongly suggests that identity problems are a potential risk factor for developing NSSI. Moreover, despite theoretical suggestions that mentalization may be associated with NSSI, few studies have directly assessed the relationship between mentalization skills and NSSI. Identity diffusion and mentalization are considered transdiagnostic factors that may contribute to the development of NSSI. However, to our knowledge, no study has yet evaluated the relationship between NSSI and mentalization, identity diffusion, and psychopathology in adolescents as a whole. The purpose of this study was to address this empirical gap by cross‐sectionally assessing the relationship among mentalization, identity diffusion, psychopathology and NSSI. To identify variables associated with NSSI, study variables were compared between clinical NSSI cases and two control groups (psychiatric patients without NSSI and healthy controls). In addition, a multivariate logistic regression model was used to examine which of the NSSI‐related variables had a unique effect on the presence of NSSI in the whole sample. The hypotheses of the study were that mentalization would be impaired and identity diffusion would be higher in adolescents with NSSI compared to both psychiatric controls and healthy controls. Additionally, it was hypothesized that there would be a negative correlation between mentalization capacity and both identity diffusion and psychopathology.

## 2 METHOD

### 2.1 Participants & procedures

The study was planned as a clinical case‐controlled, cross‐sectional study. The study case group consists of psychiatric outpatients with NSSI. There are two control groups in the study: a psychiatric clinical control group without NSSI and a healthy nonclinical control group. All consecutive admissions (*N* = 190) to the outpatient clinic of University Department of Child and Adolescent Psychiatry were approached to participate in the study between June 1, 2022 and August 31, 2022. 105 of the 190 outpatients gave consent to participate in the study. Among the psychiatric outpatients, those who met the study criteria and had engaged in NSSI behaviors in the past year were included in the NSSI group, and those without NSSI were included in the psychiatric control group. The inclusion criteria of the study were as follows: Adolescents aged 12 to 18 years without disorders of intellectual development, autism spectrum disorder, psychotic symptoms and neurological disease. Of 105 adolescents, four were excluded from the study due to exclusion criteria including IQ < 70, psychosis, and a diagnosis of autism spectrum disorder. Of the 101 psychiatric outpatients, 56 who had engaged in NSSI behavior in the past year were included in the NSSI group. The psychiatric control group consisted of 45 adolescents with outpatient psychiatric follow‐up without NSSI.

To reach the healthy control group, the study was announced at the university hospital through newsletters, bulletin boards in clinical departments, and electronic communication channels accessible to hospital staff. 63 adolescents who volunteered to participate in the study were evaluated by a child psychiatrist using a psychiatric interview based on the Diagnostic and Statistical Manual of Mental Disorders, Fifth Edition (DSM‐5). Nine adolescents were excluded from the study, because they were diagnosed with psychiatric disorders [two with attention deficit hyperactivity disorder (ADHD), one with ADHD and anxiety disorder, one with binge eating disorder, one with generalized anxiety disorder (GAD), two with depression, and two with social anxiety disorder (SAD)]. Two adolescents with a history of NSSI were excluded. The healthy control group consisted of 52 adolescents without any psychiatric diagnosis and history of NSSI.

This study was approved by Non‐Interventional Clinical Research Ethics Committee with the date 19.04.2022 and decision number 2022/11‐19. Informed consent for all participants was first obtained from the parents and, if they consented, from the adolescent in person. Adolescents were then assessed by the first author following the instructions provided with each assessment tool.

### 2.2 Measures

#### 2.2.1 Sociodemographic questionnaire

This form includes age, gender, educational status, health status, number of siblings, health status of parents and siblings, presence of psychiatric disorder of parents and siblings, family structure and parents’ relationship status.

#### 2.2.2 Screening questionnaire form of NSSI

This form was created to determine the presence and frequency of NSSI in the past year (Zeki & Çuhadaroğlu, 2012).

#### 2.2.3 Inventory of Statements About Self‐injury (ISAS)

The ISAS was developed by Klonsky and Glen (2009). It consists of two parts; the first part asks about the lifetime frequency of 12 types of self‐injurious behaviors performed intentionally and without suicidal intent, and the second part asks about the different functions of NSSI. In this study, only the first part was used to evaluate NSSI characteristics. The ISAS is a valid and reliable psychometric instrument for assessing NSSI in Turkish adolescents, with a Cronbach's α = .79 for the first part (Bildik et al., 2013). Internal consistency in the current study was sufficient, with a Cronbach's α = .67.

#### 2.2.4 Movie for the Assessment of Social Cognition (MASC)

The MASC is a computerized test developed by Dziobek et al. (2006) to assess theory of mind or mentalizing abilities. Studies have shown that the MASC is an appropriate test for assessing mentalization skills in adolescents (Gambin et al., 2015; Quek et al., 2018; Sharp et al., 2011). The test provides dialogues of young people from everyday life, through a 15‐min movie and is based on estimating the mental states of the characters. The task consists of 45 multiple‐choice questions with four options of patterns of mentalization: a) *correct mentalizing*, indicating a balanced inference of others’ mental states b) *hypermentalizing*, indicating an excessive attribution of mental states c) *undermentalizing*, indicating a reduced use of others’ mental states d) *no‐mentalizing*, indicating a complete absence of mentalizing. MASC total score is calculated as the total number of correct mentalizing answers to all mentalization questions (range: 0‐45). Additionally, MASC error types (hypermentalizing, no‐mentalizing, and undermentalizing) are calculated separately by summing the number of each type.

For this study, MASC was translated from English to Turkish with the permission of Isabel Dziobek. Turkish dubbing of the movie videos used in the computerized test was done by professional actors in the sound recording studio. After the sound recording phase, the audio‐video combination (dubbing) was made by professionals in the recording studio by adapting the lip movements of the actors in the movie to the same model as the English version. The Turkish version was first applied to 10 healthy adolescents for a pilot study and feedback was received from them about the dubbing quality and the ease of understanding of the test. All of them stated that the sound quality was good, and they could understand easily. The psychometric properties of the MASC have demonstrated good internal consistency, with a Cronbach's α = .84 (Dziobek et al., 2006). Internal consistency in the current study was sufficient, with a Cronbach's α = .71.

#### 2.2.5 Assessment of IDENTITY Development in Adolescence (AIDA)

The AIDA (Assessment of Identity Development in Adolescence) was developed by Goth et al. (2012) to assess identity development along a complex dimension, ranging from “Identity Integration” to “Identity Diffusion”. The measure consists of 58 items and each item is rated on a five‐point Likert scale ranging from 0 (*no)* to 4 (*yes*). The total score obtained by summing the scores of each item reflects a higher degree of identity diffusion. The Turkish version of the questionnaire demonstrated excellent internal consistency for the total diffusion score, with a Cronbach's α = .93 (Çuhadaroğlu et al., 2023). In the current study, internal consistency was also excellent, with a Cronbach's α = .95.

#### 2.2.6 The Youth Self‐Report (YSR)

The YSR was developed by Achenbach (1991) to provide a standardized way to assess adolescents’ areas of competence and problem behaviors on a self‐report basis. Each item is rated on a three‐point Likert scale ranging from 0 (*not relevant*) to 2 (*very relevant*). A T score on the YSR with a cut‐off of >65 is recommended for a higher risk of psychopathology. The scale measures a total problem T score of general psychiatric functioning and two broad subscales of externalizing and internalizing behavior problems. Externalizing subscale measures aggressive behavior and rule‐breaking behavior; and internalizing one covers anxious/depressed, withdrawn/depressed, and somatic complaints. The psychometric properties of the YSR have demonstrated good internal consistency among Turkish adolescents, with a Cronbach's α = .84 for the total problem score (Erol et al., 1998). In the current study, this questionnaire was scored electronically, so item‐level data were not available for analysis of internal consistency.

### 2.3 Data analyses

Statistical analysis was performed using the SPSS 26 software package. First, descriptive statistics and bivariate relations were used to examine the characteristics and relations of the study variables. The suitability of the variables for normal distribution was tested by visual (histogram and probability plots) and analytical methods (Kolmogorov‐Smirnov/Shapiro‐Wilk tests). Bivariate relations between the study variables were examined using Pearson or Spearman, χ2, t‐test, or Mann‐ Whitney U test. Analysis of variance was performed with ANOVA or Kruskal‐Wallis. Effect size was reported as partial eta‐squared for ANOVA. Post Hoc Analysis with Bonferroni adjustment is used to identify which groups are significantly different from each other. A binary logistic regression analysis was used to examine the effect of independent variables on the dependent variable (NSSI). Independent variables associated with NSSI were concurrently entered into a multivariate logistic regression to determine variables that had a unique impact on NSSI.

## 3 RESULTS

### 3.1 Sociodemographic characteristics

A total of 153 adolescents aged 12 to 18 years, 56 (36.6%) psychiatric outpatients with NSSI, 45 (29.4%) psychiatric outpatients without NSSI, and 52 healthy controls (34.0%) were included. The total sample consisted of 76.5% girls and 23.5% boys.

Sociodemographic characteristics and the main study variables were compared between groups (Table 1). The ratio of girls in the NSSI group was significantly higher compared to the healthy control group (*χ2* = 8.72, *p* = .013). The presence of a maternal psychiatric disorder was found to be significantly higher in the NSSI group than in the other groups (*χ2* = 5.28, *p* =.022). Maternal age (*F* = 2.00, *p* = .131), paternal age (*F* = 0.59, *p* = .552), maternal medical disease (*χ2* = 0.08, *p* = .962), paternal medical disease (*χ2* = 1.86, *p* = .395), presence of paternal psychiatric disorder (*χ2* = 4.78, *p* = .079), family structure (*χ2* = 0.02, *p* = .988), parents’ relationship status (*χ2* = 11.34, *p* = .057), and number of children (*H* = 1.69, *p* = .728) did not differ between three groups.

**Table 1:** Comparison of sociodemographic characteristics and the main study variables between groups.

	NSSI^1^ *n* = 56 (36,6%)		Psychiatric control^2^ *n* = 45 (29,4%)		Healthy control^3^ *n* = 52 (34%)		*p*	Group Diffrence (1,2,3)
	*n*	%	*n*	%	*n*	%		
Gender								
Girls	49	87.5	35	77.8	33	63.5	.013a	1 > 3
Boys	7	12.5	10	22.2	19	36.5		

	M	SD	M	SD	M	SD		
Age	15.3	1.4	15.9	1.4	15.7	1.2	.062b	
Education (years)	10.1	1.2	10.8	1.3	10.5	1.1	.014b	1 < 2
MASC								
MASC Total	25.7	4.7	23.3	4.9	28.4	2.2	.008b	1 < 3
Hypermentalizing	10.3	3.6	9.2	3.3	9.8	2.6	.226b	
Undermentalizing	5.2	3.1	5.5	2.8	4.4	1.9	.152b	
No‐mentalizing	3.8	2.8	2.8	2.1	2.3	1.5	.014b	1 > 3; 1 > 2
MASC_control	4.1	1.1	4.5	1.1	4.3	1.1	.144b	
AIDA								
Identity diffusion	136.6	35.3	91.7	40.9	75.6	33.6	< .001c	1 > 3; 1 > 2
YSR								
Internalizing	70.6	8.5	63.2	10.8	58.6	6.9	< .001c	1 > 2; 1 > 3
Externalizing	64.2	7.5	62.3	7.2	55.8	5.5	< .001b	1 > 3; 2 > 3
Total problem	69.4	7.4	63.2	8.3	56.6	7.0	< .001c	1 > 2 > 3

Abbreviations: AIDA, Assessment of Identity Development in Adolescence; MASC, Movie for the Assessment of Social Cognition; YSR, Youth Self Report

^1^ Nonsuicidal self‐injury group,

^2^ Psychiatric control group,

^3^ Healthy group

^a^ Pearson Chi‐Square,

^b^ Kruskal Wallis Test,

^c^ One Way ANOVA

### 3.2 Characteristics of NSSI behavior

Among 56 adolescents with NSSI, 26 (46.4%) reported engaging in NSSI behaviors one to five times, and 30 (53.6%) reported engaging in these behaviors five or more times over a one‐year period. There was no significant relationship between gender and the frequency of NSSI (*χ2* = 2.57, *p* = .419). The mean age of onset of NSSI was 12.57 years (SD = 2.11, range = 5‐17). There was no gender difference in the age of onset of NSSI (*Z* = 0.75, *p* = .481). The most common type of NSSI method was cutting (75%), followed by banging/hitting (66.1%) and wound picking (66.1%). There was no significant difference in any of the NSSI methods by gender. 32.1% of adolescents with NSSI reported that they felt physical pain during NSSI, 42.9% reported that they sometimes felt physical pain and 25% did not. Among adolescents with NSSI, 87.5% reported being alone during NSSI, 75% reported that there was less than 1 hour between the urge to self‐injure and the act, and 57.1% wanted to stop self‐injuring.

### 3.3 Comparison of mentalizing, identity diffusion and psychopathology among groups

Comparisons of MASC, AIDA and YSR between groups were shown in Table 1. The Kruskal‐Wallis H test showed a significant difference between the groups on the MASC total scores (*H* = 9.65, *p* = .008). In the post hoc pairwise comparison using the Bonferroni‐corrected alpha level of 0.017, MASC total scores were significantly lower in the NSSI group than in the healthy control group (*Z* = −2.92, *p* = .011). No‐mentalizing differentiated the NSSI group (*H* = 8.48, *p* = .014), but there was no significant difference between the non‐NSSI groups (*Z* = 0.507, p = 1.00). There was no difference between the three groups in terms of under‐mentalizing (*H* = 3.77, *p* = .152) and hypermentalizing (*H* = 2.98, *p* = .226). Thus, NSSI group was differentiated from the two control groups by their low mentalizing capacities (higher no mentalizing).

Identity diffusion score was significantly different between groups (*F* = 41.53, *p* < .001, *η²* = 0.36). NSSI group had significantly higher identity diffusion scores than the psychiatric control group and the healthy control group, respectively (*p* < .001; *p* < .001). There was no difference between the psychiatric control and the healthy control (*p* = .083). Identity diffusion differentiated the NSSI group from the others.

There was a significant effect of group on internalizing (*F* = 25.87, *p* <.001, *η2* = 0.26), externalizing (*H* = 36.76, *p* < .001) and total problems scores (*F* = 38.28, *p* < .001, *η2* = 0.34). Internalizing problems were significantly higher in the NSSI group compared to both the psychiatric control group and the healthy control group, respectively (*p* = .001; *p* < .001). There was no significant difference in externalizing problem scores between the NSSI group and the psychiatric control group (*Z* = 1.30, *p* = .058), but it was significantly lower in the healthy control group compared to NSSI (*Z* = 5.85, *p* <.001) and psychiatric control groups (*Z* = 4.25, *p* < .001). On YSR, internalizing and total problem differentiated the NSSI group.

### 3.4 The relationship between mentalization, identity diffusion and psychopathology

The results of the correlation analysis of mentalization, identity diffusion and psychopathology in the whole study group were given in Table 2. There was a significant relationship between age and MASC total scores (*r* = .232, *p* = .004). Identity diffusion was associated with all types of YSR problem behavior. MASC total score was significantly negatively related to identity diffusion (*r* = −0.227, *p* = .005). There was a significant negative relationship between MASC total scores and internalizing problems (*r* = −0.181, *p* = .025). No significant relationship was found between MASC total scores and externalizing problems (*r* = −0.070, *p* = .389). MASC total scores showed a negative significant relationship with total problems (*r* = −0.210, *p* = .009). In summary, MASC total scores increased by age, decreased by identity diffusion score, and decreased by internalizing and total problems.

**Table 2:** Bivariate correlation between the main study variables (N:153).

Variable	1	2	3	4	5	6	7	8	9	10	11	12
1. Age	‐											
2. Gender	0.02	‐										
3. Presence of NSSI	−0.20	−0.20*	‐									
4. MASC total	.23**	0.05	−0.25**	‐								
5. Hypermentalizing	−0.05	0.12	0.11	−0.54**	‐							
6. Undermentalizing	−0.02	−0.13	0.01	−0.37**	−0.36**	‐						
7. No‐mentalizing	−0.10	−0.15	0.22**	−0.47**	−0.17*	0.17*	‐					
8. MASC_control	−0.13	−0.05	−0.14	0.12	−0.05	−0.03	−0.09	‐				
9. Identity diffusion score	−0.12	−0.21**	0.58**	−0.23**	0.13	0.05	0.16	0.01	‐			
10. Internalizing	−0.04	−0.03	0.47**	−0.18*	0.09	0.06	0.11	0.01	0.76**	‐		
11. Externalizing	<−0.01	0.05	0.35**	−0.07	0.13	−0.02	−0.06	−0.07	0.47**	0.40**	‐	
12. Total problem	−0.06	−0.01	0.53**	−0.21**	0.14	0.06	0.08	−0.03	0.79**	0.85**	0.74**	‐

*Note*: Spearman Rho test

* *p* <.05;

** *p* < .01

### 3.5 Regression analyses

In the total sample (*N* = 153), a multivariate logistic regression model was applied using the enter method to determine the specificity of the variables related to NSSI (Table 3). The variables that remained significantly associated with the presence of NSSI were the presence of a maternal psychiatric disorder (*p* = .019, *OR* = 5.21), identity diffusion score of AIDA (*p* = .007, *OR* = 1.02), no‐mentalizing score (*p* = .049, *OR* = 1.28), and total problem T score of YSR (*p* = .009, *OR* = 1.12).

**Table 3:** Logistic regression assessing the risk factors for the presence of NSSI (N:153).

	B	SE	Wald	OR	95% CI		*p*
					LL	UL	
Constant	−6.916	3.938	3.084	‐	‐	‐	.079
Age (year)	−0.291	.167	3.034	0.748	0.54	0.04	.082
Gender	.765	.627	1.488	0.465	0.14	1.59	.222
Presence of maternal psychiatric disorder	1.650	.706	5.467	5.209	1.31	20.78	.019
Identity diffusion score of AIDA	.022	.008	7.403	1.022	1.01	1.04	.007
Total score of MASC	.011	.069	.023	1.011	0.88	1.16	.879
No‐mentalizing score of MASC	.249	.126	3.890	1.282	1.00	1.64	.049
Total problem T score of YSR	.112	.043	6.907	1.119	1.03	1.22	.009

*Note*: Hosmer and Lemeshow goodness of fit with good results (*χ2* = 8.67, *p* = .371); *R* ^*2*^ (Cox and Snell) = 0.405 and *R* ^*2*^ (Nagelkerte) = 0.554

Abbreviations: AIDA, Assessment of Identity Development in Adolescence; MASC, Movie for the Assessment of Social Cognition; YSR, Youth Self Report

## 4 DISCUSSION

This study is the first to examine the impact of mentalization, identity diffusion, and psychopathology on NSSI from a broader trans‐diagnostic perspective. Studies highlight that identity issues may be a significant risk factor for the development of NSSI. Additionally, while there are theoretical suggestions linking mentalization to NSSI, only a limited number of studies have directly examined this relationship. Both identity diffusion and mentalization may act as transdiagnostic factors influencing the development of NSSI. Therefore, this study aimed to provide a comprehensive assessment of the interplay among mentalization skills, identity diffusion, psychopathology, and NSSI in adolescents.

The results of this study revealed that MASC total scores were significantly lower and no‐mentalizing scores were significantly higher in the NSSI group, confirming our hypothesis that mentalization would be more impaired in adolescents with NSSI. Consistent with our findings, Laghi et al. (2016) reported that female inpatient adolescents with NSSI had lower levels of mentalization. These findings are reasonable, given that mentalization is a key element in managing emotions within social situations. The ability to understand the mental states of others seems to be of crucial importance for successful social interactions and for adequate psychosocial functioning (Ballespí et al., 2021; Brackett et al., 2006). Nock (2010) also posited that in the context of interpersonal difficulties, NSSI serves to regulate social situations and manage interpersonal relationships. When individuals struggle to recognize their emotional needs, they may feel isolated and unable to articulate their distress. In such circumstances, NSSI may emerge to achieve temporary relief from overwhelming emotions.

Consistent with previous studies showing an association between identity diffusion and NSSI (Claes et al., 2014; Claes et al., 2015; Gandhi et al., 2016; Luyckx et al., 2015a, 2015b), our results indicated that identity diffusion significantly increased the risk of engaging in NSSI. Theoretically, individuals may engage in self‐harming behaviors to escape the negative emotions and thoughts associated with identity diffusion. In addition, some individuals may regard these behaviors as a source for achieving a pseudo‐identity (e.g., I am a self‐injurer) (Breen et al., 2013; Gandhi et al., 2016). As identity diffusion increases, NSSI may a highly maladaptive way to maintain a coherent sense of self. In contrast, healthy resolution of identity development issues may act as a protective factor against NSSI, enabling individuals to maintain a stable self‐concept and develop effective coping strategies.

The results of this study demonstrated a significant relationship between identity diffusion and mentalization capacity. The capacity to understand others’ mental states plays a crucial role in identity development by shaping how individuals perceive themselves in relation to others and the social world around them (Fonagy & Target, 1996). From a mentalizing perspective, the sense of self‐coherence is a product of the capacity for mentalization and the impairments in mentalizing are considered to be leading to identity diffusion (Bargh, 2014; Fonagy & Bateman, 2019). Identity diffusion has been shown in several studies to reflect borderline personality features in clinical and nonclinical adolescent samples (Goth et al., 2012; Rivnyák et al., 2021). Many studies using the MASC have found an association between hypermentalizing and borderline personality traits (Quek et al., 2018; Sharp et al., 2016). However, this study found no significant relationship between hypermentalizing and identity diffusion. Another study of female adolescents in Egypt found a significant association with undermentalizing, but not with hypermentalizing (Goueli et al., 2020). Differences in findings across studies examining the relationship between borderline personality traits and mentalization may be due to cultural factors and the use of different measures of borderline personality features.

Existing literature acknowledges a bidirectional relationship between mentalization and psychopathology, suggesting that mentalization problems may increase the risk of developing psychological disorders, while existing psychopathological conditions may also disrupt mentalization processes (Fonagy & Allison, 2012; Luyten et al., 2020). The study findings indicated that mentalization capacity was associated with psychopathology symptoms. In contrast, a study of nonclinical adolescents found no significant relationship between mentalization and psychopathology symptoms. Nevertheless, it has been suggested that mentalization may enhance mental health by fostering resilience and well‐being beyond symptomatic outcomes (Ballespí et al., 2018). Contrary to previous studies that found a significant relationship between hypermentalizing and internalizing problems (Gambin et al., 2015; Poznyak et al., 2019), this study found no significant relationship between hypermentalizing and internalizing problems. These findings point to the complexity of the relationship between mentalization and psychopathology and suggest that further research might explore the underlying mechanisms and contextual and cultural factors that influence these dynamics.

Regarding NSSI characteristics, the high rate of girls in the NSSI group is consistent with studies showing a higher prevalence of NSSI in girls (Muehlenkamp & Gutierrez, 2007; Xiao et al., 2022). The finding that cutting is the most commonly reported method of NSSI is consistent with other studies (Laye‐Gindhu & Schonert‐Reichl, 2005; Nock et al., 2006), too. No gender differences were observed in either the frequency or method of NSSI, contrary to studies suggesting gender differences (Bresin & Schoenleber, 2015; Muehlenkamp & Gutierrez, 2007; Xiao et al., 2022).

An additional important finding of this study was that the presence of a maternal psychiatric disorder increased the risk of NSSI, independent of other variables. Emotion regulation skills are acquired based on the attachment relationship established with the mother in early childhood (Bowlby, 1988; Brumariu et al., 2012). It may be interpreted that maternal psychiatric disorder may be a risk factor for the development of NSSI later in life, as it may negatively affect the attachment relationship and thus the development of mentalization and emotion regulation skills.

This is the first study to report an association between NSSI and no‐mentalizing independent of other variables (age, gender, identity diffusion, total psychopathology symptoms, and presence of maternal psychiatric disorder). Current findings support the Mentalization‐Based Therapy model, which suggests that a lack of mentalization increases the likelihood of using physical actions, such as NSSI, as a coping mechanism for intolerable situations (Bateman & Fonagy, 2010). Increasing mentalization capacity can also potentially reduce NSSI by enhancing individuals’ ability to understand and interpret their own emotional states and those of others (Rossouw et al., 2020). By improving mentalization skills, adolescents may become more capable of recognizing their emotional needs, leading to healthier emotional regulation strategies. Moreover, as adolescents develop a better understanding of social cues and interpersonal dynamics, they are likely to foster more meaningful relationships and gain support from peers and family. Such social connections can provide alternative coping mechanisms and emotional support, making it less likely for adolescents to resort to NSSI. Thus, integrating mentalization interventions into therapeutic approaches may hold promise for reducing NSSI.

This study has several strengths that significantly enhance our understanding of the trans‐diagnostic and developmental factors associated with NSSI. First, current findings reveal a significant relationship between mentalization, identity diffusion, psychopathology, and NSSI, which may have important implications for understanding of NSSI among adolescents from a comprehensive perspective and the developing clinical interventions. A notable strength of this study is also the adaptation of the MASC into Turkish, a comprehensive instrument for assessing mentalization skills in adolescents. Additionally, the inclusion of both clinical and nonclinical control groups further strengthens the study by providing robust evidence for identifying discriminative risk factors for NSSI.

Nevertheless, this study has some limitations. Firstly, the cross‐sectional design makes it difficult to establish causal relationships. Therefore, future longitudinal studies may provide further insight into understanding how mentalization and identity diffusion interact to confer risk for NSSI in adolescents. Additionally, the relationship between the measured variables is likely to be bidirectional. Originally it was planned to do a SEM analysis for the data to test the interactional effects of the variables in a developmental model for NSSI; however, our sample was not sufficient to carry on this modeling analysis. Even though we couldn't test a model, considering the theoretical knowledge we have from the literature and the results of the previous studies and the current one, we may conclude that NSSI is closely related to, and may be the result of the negative developmental characteristics (e.g., low mentalizing capacity and identity diffusion) which makes the emotional regulation difficult under the challenges and risks of adolescence. Here low mentalizing capacity and identity diffusion may be enhancing each other's effect when together, too. Since emotion regulation difficulty is repeatedly found to be related with NSSI in studies, low mentalizing capacity along with identity diffusion, and/or in presence of psychopathology may easily activate NSSI behavior as a means of maladaptive emotion regulation.

## 5 CONCLUSIONS

The results of this study indicated that adolescents with NSSI had significantly lower mentalizing capacity and higher rates of no‐mentalizing errors. Mentalization deficits may underlie social and emotional functioning problems, leading adolescents to engage in NSSI as a means of regulating their internal responses or influencing their social environment. Enhancing mentalization capacity may also play an important role in the prevention and reduction of NSSI among adolescents. Furthermore, considering the findings on the relationship between mentalizing capacity and identity diffusion, interventions focusing on increasing mentalizing ability may be beneficial in reducing borderline features. In addition, evaluating identity development processes in adolescents and supporting them in managing difficulties during this phase may help prevent identity diffusion, thereby playing a protective role against NSSI. Overall, the current findings highlight that a multidimensional approach incorporating assessments of mentalization and identity diffusion may contribute to a more comprehensive understanding of NSSI and the development of clinical interventions for adolescents with NSSI.

## CONFLICT OF INTEREST STATEMENT

The authors declare no conflicts of interest.

## ETHICS STATEMENT

This study was approved by Hacettepe University Non‐Interventional Clinical Research Ethics Committee with the date 19.04.2022 and decision number 2022/11‐19.

## Data Availability

The data that support the findings of this study are available from the corresponding author upon reasonable request.

## References

[jad12422-bib-0001] Achenbach, T. M. (1991). Manual for the Youth Self‐Report and 1991 Profile. University of Vermont Department of Psychiatry.

[jad12422-bib-0003] Andrews, J. L. , Ahmed, S. P. , & Blakemore, S. J. (2021). Navigating the social environment in adolescence: The role of social brain development. Biological Psychiatry, 89(2), 109–118. 10.1016/j.biopsych.2020.09.012 33190844

[jad12422-bib-0004] Ballespí, S. , Vives, J. , Debbané, M. , Sharp, C. , & Barrantes‐Vidal, N. (2018). Beyond diagnosis: Mentalization and mental health from a transdiagnostic point of view in adolescents from non‐clinical population. Psychiatry Research, 270, 755–763. 10.1016/j.psychres.2018.10.048 30551321

[jad12422-bib-0005] Ballespí, S. , Vives, J. , Sharp, C. , Chanes, L. , & Barrantes‐Vidal, N. (2021). Self and other mentalizing polarities and dimensions of mental health: Association with types of symptoms, functioning and Well‐Being. Frontiers in Psychology, 12, 566254. 10.3389/fpsyg.2021.566254 33613372 PMC7886987

[jad12422-bib-0006] Bargh, J. A. (2014). Our unconscious mind. Scientific American, 310, 30–37. 10.1038/scientificamerican0114-30 24616968

[jad12422-bib-0007] Bateman, A. , & Fonagy, P. (2010). Mentalization based treatment for borderline personality disorder. World Psychiatry, 9(1), 11–15. 10.1002/j.2051-5545.2010.tb00255.x 20148147 PMC2816926

[jad12422-bib-0008] Benzi, I. , & Madeddu, F. (2017). Development of personality disorders: Identity as a key process. International Journal of Psychology & Behavior Analysis, 3, 124. 10.15344/2455-3867/2017/124

[jad12422-bib-0009] Bildik, T. , Somer, O. , Kabukçu Başay, B. , Başay, Ö. , & Özbaran, B. (2013). [The validity and reliability of the Turkish version of the inventory of statements about self‐injury]. Turk Psikiyatri Dergisi = Turkish Journal of Psychiatry, 24(1), 49–57. 23446540

[jad12422-bib-0010] Bowlby, J. (1988). A secure base: *Parent‐child attachment and healthy human development* . Basic Books.

[jad12422-bib-0011] Brackett, M. A. , Rivers, S. E. , Shiffman, S. , Lerner, N. , & Salovey, P. (2006). Relating emotional abilities to social functioning: A comparison of self‐report and performance measures of emotional intelligence. Journal of Personality and Social Psychology, 91(4), 780–795. 10.1037/0022-3514.91.4.780 17014299

[jad12422-bib-0012] Breen, A. V. , Lewis, S. P. , & Sutherland, O. (2013). Brief report: Non‐suicidal self‐injury in the context of self and identity development. Journal of Adult Development, 20(1), 57–62. 10.1007/s10804-013-9156-8

[jad12422-bib-0013] Bresin, K. , & Schoenleber, M. (2015). Gender differences in the prevalence of nonsuicidal self‐injury: A meta‐analysis. Clinical Psychology Review, 38, 55–64. 10.1016/j.cpr.2015.02.009 25795294

[jad12422-bib-0014] Brown, R. C. , & Plener, P. L. (2017). Non‐suicidal self‐injury in adolescence. Current Psychiatry Reports, 19(3), 20. 10.1007/s11920-017-0767-9 28315191 PMC5357256

[jad12422-bib-0015] Brumariu, L. E. , Kerns, K. A. , & Seibert, A. (2012). Mother‐child attachment, emotion regulation, and anxiety symptoms in middle childhood. Personal Relationships, 19(4), 569–585. 10.1111/j.1475-6811.2011.01379.x

[jad12422-bib-0016] Claes, L. , Luyckx, K. , & Bijttebier, P. (2014). Non‐suicidal self‐injury in adolescents: Prevalence and associations with identity formation above and beyond depression. Personality and Individual Differences, 61–62, 101–104. 10.1016/j.paid.2013.12.019

[jad12422-bib-0017] Claes, L. , Luyckx, K. , Bijttebier, P. , Turner, B. , Ghandi, A. , Smets, J. , Norre, J. , Van Assche, L. , Verheyen, E. , Goris, Y. , Hoksbergen, I. , & Schoevaerts, K. (2015). Non‐Suicidal Self‐Injury in patients with eating disorder: Associations with identity formation above and beyond anxiety and depression. European Eating Disorders Review, 23, 119–125. 10.1002/erv.2341 25504562

[jad12422-bib-0018] Crocetti, E. , Luyckx, K. , Scrignaro, M. , & Sica, L. S. (2011). Identity formation in Italian emerging adults: A cluster‐analytic approach and associations with psychosocial functioning. European Journal of Developmental Psychology, 8, 558–572. 10.1080/17405629.2011.576858

[jad12422-bib-0019] Çuhadaroğlu, F. Ç. , Tüzün, Z. , Akdemir, D. , & Özdemir DF ve Ataman, E. (2023). Culture adapted version Turkish of the self‐report questionnaire AIDA (Assessment of Identity Development in Adolescence, authors Goth & Schmeck)‐Short manual. Academic‐tests [Turkish]. https://academic-tests.com/aida/#aida1218tr

[jad12422-bib-0020] Desatnik, A. , Bird, A. , Shmueli, A. , Venger, I. , & Fonagy, P. (2023). The mindful trajectory: Developmental changes in mentalizing throughout adolescence and young adulthood. PLoS One, 18(6), e0286500. 10.1371/journal.pone.0286500 37343006 PMC10284399

[jad12422-bib-0021] Dziobek, I. , Fleck, S. , Kalbe, E. , Rogers, K. , Hassenstab, J. , Brand, M. , Kessler, J. , Woike, J. K. , Wolf, O. T. , & Convit, A. (2006). Introducing MASC: A movie for the assessment of social cognition. Journal of Autism and Developmental Disorders, 36(5), 623–636. 10.1007/s10803-006-0107-0 16755332

[jad12422-bib-0022] Erikson, E. H. (1968). Identity: Youth and crisis. W.W. Norton & Company.

[jad12422-bib-0023] Erol, N. , Kılıç, C. , Ulusoy, M. , & Kececi, M. (1998). Mental health profile in Turkey: A main report (pp. 77–93). Turkish Republic, Ministry of Health

[jad12422-bib-0024] Fonagy, P. , & Allison, E. (2012). What is mentalization? The concept and its foundations in developmental research. In N. Midgley , & I. Vrouva eds., Minding the child (pp. 11–34). Routledge.

[jad12422-bib-0025] Fonagy, P. , & Bateman, A. (2019). Handbook of mentalizing in mental health practice. American Psychiatric Association Publishing.

[jad12422-bib-0026] Fonagy, P. , & Target, M. (1996). Mentalization and the development of the self, Handbook of self and identity (pp. 120–140). Guilford Press.

[jad12422-bib-0027] Gambin, M. , Gambin, T. , & Sharp, C. (2015). Social cognition, psychopathological symptoms, and family functioning in a sample of inpatient adolescents using variable‐centered and person‐centered approaches. Journal of Adolescence, 45, 31–43. 10.1016/j.adolescence.2015.08.010 26356807

[jad12422-bib-0028] Gandhi, A. , Luyckx, K. , Baetens, I. , Kiekens, G. , Sleuwaegen, E. , Berens, A. , Maitra, S. , & Claes, L. (2018). Age of onset of non‐suicidal self‐injury in Dutch‐speaking adolescents and emerging adults: An event history analysis of pooled data. Comprehensive Psychiatry, 80, 170–178. 10.1016/j.comppsych.2017.10.007 29121554

[jad12422-bib-0029] Gandhi, A. , Luyckx, K. , Goossens, L. , Maitra, S. , & Claes, L. (2016). Sociotropy, autonomy, and non‐suicidal self‐injury: The mediating role of identity confusion. Personality and Individual Differences, 99, 272–277. 10.1016/j.paid.2016.05.040

[jad12422-bib-0030] Gandhi, A. , Luyckx, K. , Maitra, S. , Kiekens, G. , Verschueren, M. , & Claes, L. (2017). Directionality of effects between non‐suicidal self‐injury and identity formation: A prospective study in adolescents. Personality and Individual Differences, 109, 124–129. 10.1016/j.paid.2017.01.003

[jad12422-bib-0031] Glenn, C. R. , & Klonsky, E. D. (2013). Nonsuicidal self‐injury disorder: An empirical investigation in adolescent psychiatric patients. Journal of Clinical Child and Adolescent Psychology: The Official Journal for the Society of Clinical Child and Adolescent Psychology, American Psychological Association, Division 53, 42(4), 496–507. 10.1080/15374416.2013.794699 23682597 PMC4433043

[jad12422-bib-0032] Goth, K. , Foelsch, P. , Schlüter‐Müller, S. , Birkhölzer, M. , Jung, E. , Pick, O. , & Schmeck, K. (2012). Assessment of identity development and identity diffusion in adolescence: Theoretical basis and psychometric properties of the self‐report questionnaire AIDA. Child and adolescent psychiatry and mental health, 6(1), 27. 10.1186/1753-2000-6-27 22812911 PMC3485126

[jad12422-bib-0033] Goueli, T. , Nasreldin, M. , Madbouly, N. , Dziobek, I. , & Farouk, M. (2020). Social cognition in adolescent females with borderline personality traits. Psychology and Psychotherapy: Theory, Research and Practice, 93(4), 739–753. 10.1111/papt.12257 31692159

[jad12422-bib-0034] Gratz, K. L. , Dixon‐Gordon, K. L. , Chapman, A. L. , & Tull, M. T. (2015). Diagnosis and characterization of DSM‐5 nonsuicidal self‐injury disorder using the clinician‐administered nonsuicidal self‐injury Disorder Index. Assessment, 22(5), 527–539. 10.1177/1073191114565878 25604630 PMC5505727

[jad12422-bib-0035] Guerry, J. D. , & Prinstein, M. J. (2010). Longitudinal prediction of adolescent nonsuicidal self‐injury: Examination of a cognitive vulnerability‐stress model. Journal of Clinical Child and Adolescent Psychology: The Official Journal for the Society of Clinical Child and Adolescent Psychology, American Psychological Association, Division 53, 39(1), 77–89. 10.1080/15374410903401195 20390800 PMC4626882

[jad12422-bib-0037] Jacobson, C. M. , & Gould, M. (2007). The epidemiology and phenomenology of non‐suicidal self‐injurious behavior among adolescents: A critical review of the literature. Archives of Suicide Research, 11(2), 129–147. 10.1080/13811110701247602 17453692

[jad12422-bib-0038] Kahveci, N. , & Cetin, F. (2020). *Evaluation of mindfulness and family attitudes in adolescents with and without nonsuicidal self‐injurious behavior* [Unpublished specialization thesis]. Hacettepe University.

[jad12422-bib-0039] Kernberg, P. F. , Weiner, A. S. , & Bardenstein, K. K. (2000). Personality disorders in children and adolescents. Basic Books.

[jad12422-bib-0040] Kerr, P. L. , Muehlenkamp, J. J. , & Turner, J. M. (2010). Nonsuicidal self‐injury: A review of current research for family medicine and primary care physicians. The Journal of the American Board of Family Medicine, 23(2), 240–259. 10.3122/jabfm.2010.02.090110 20207935

[jad12422-bib-0041] Klonsky, E. D. (2007). The functions of deliberate self‐injury: A review of the evidence. Clinical Psychology Review, 27(2), 226–239. 10.1016/j.cpr.2006.08.002 17014942

[jad12422-bib-0072] Klonsky, E. D. , & Glenn, C. R. (2009). Assessing the functions of non‐suicidal self‐injury: Psychometric properties of the inventory of statements about self‐injury (ISAS). Journal of Psychopathology and Behavioral Assessment, 31(3), 215–219. 10.1007/s10862-008-9107-z 29269992 PMC5736316

[jad12422-bib-0042] Klonsky, E. D. , Victor, S. E. , & Saffer, B. Y. (2014). Nonsuicidal self‐injury: What we know, and what we need to know. The Canadian Journal of Psychiatry, 59(11), 565–568. 10.1177/070674371405901101 25565471 PMC4244874

[jad12422-bib-0043] Koposov, R. , Stickley, A. , & Ruchkin, V. (2021). Non‐suicidal self‐injury among incarcerated adolescents: Prevalence, personality, and psychiatric comorbidity. Frontiers in Psychiatry, 12, 652004. 10.3389/fpsyt.2021.652004 34093271 PMC8170036

[jad12422-bib-0044] Laghi, F. , Terrinoni, A. , Cerutti, R. , Fantini, F. , Galosi, S. , Ferrara, M. , & Bosco, F. M. (2016). Theory of mind in non‐suicidal self‐injury (NSSI) adolescents. Consciousness and Cognition, 43, 38–47. 10.1016/j.concog.2016.05.004 27236355

[jad12422-bib-0045] Laye‐Gindhu, A. , & Schonert‐Reichl, K. A. (2005). Nonsuicidal self‐harm among community adolescents: Understanding the “whats” and “whys” of self‐harm. Journal of Youth and Adolescence, 34, 447–457. 10.1007/s10964-005-7262-z

[jad12422-bib-0046] Lewis, S. P. , & Heath, N. L. (2015). Nonsuicidal self‐injury among youth. The Journal of Pediatrics, 166(3), 526–530. 10.1016/j.jpeds.2014.11.062 25596101

[jad12422-bib-0047] Lim, K. S. , Wong, C. H. , McIntyre, R. S. , Wang, J. , Zhang, Z. , Tran, B. X. , Tan, W. , Ho, C. S. , & Ho, R. C. (2019). Global lifetime and 12‐month prevalence of suicidal behavior, deliberate self‐harm, and non‐suicidal self‐injury in children and adolescents between 1989 and 2018: A meta‐analysis. International Journal of Environmental Research and Public Health, 16(22), 4581. 10.3390/ijerph16224581 31752375 PMC6888476

[jad12422-bib-0048] Luyckx, K. , Gandhi, A. , Bijttebier, P. , & Claes, L. (2015a). Non‐suicidal self‐injury in female adolescents and psychiatric patients: A replication and extension of the role of identity formation. Personality and Individual Differences, 77, 91–96. 10.1016/j.paid.2014.12.057

[jad12422-bib-0049] Luyckx, K. , Gandhi, A. , Bijttebier, P. , & Claes, L. (2015b). Non‐suicidal self‐injury in high school students: Associations with identity processes and statuses. Journal of Adolescence, 41, 76–85. 10.1016/j.adolescence.2015.03.003 25828550

[jad12422-bib-0050] Luyten, P. , Campbell, C. , Allison, E. , & Fonagy, P. (2020). The mentalizing approach to psychopathology: State of the art and future directions. Annual review of clinical psychology, 16, 297–325. 10.1146/annurev-clinpsy-071919-015355 32023093

[jad12422-bib-0051] Muehlenkamp, J. J. , & Gutierrez, P. M. (2007). Risk for suicide attempts among adolescents who engage in non‐suicidal self‐injury. Archives of Suicide Research, 11(1), 69–82. 10.1080/13811110600992902 17178643

[jad12422-bib-0052] Nielsen, E. , Sayal, K. , & Townsend, E. (2016). Exploring the relationship between experiential avoidance, coping functions and the recency and frequency of self‐Harm. PLoS One, 11(7), e0159854. 10.1371/journal.pone.0159854 27442036 PMC4956262

[jad12422-bib-0053] Nock, M. , JOINERJR, Jr. T. , Gordon, K. , Lloyd‐Richardson, E. , & Prinstein, M. (2006). Non‐suicidal self‐injury among adolescents: Diagnostic correlates and relation to suicide attempts. Psychiatry Research, 144(1), 65–72. 10.1016/j.psychres.2006.05.010 16887199

[jad12422-bib-0054] Nock, M. K. (2010). Self‐injury. Annual review of clinical psychology, 6, 339–363. 10.1146/annurev.clinpsy.121208.131258 20192787

[jad12422-bib-0055] Plener, P. L. , Schumacher, T. S. , Munz, L. M. , & Groschwitz, R. C. (2015). The longitudinal course of non‐suicidal self‐injury and deliberate self‐harm: A systematic review of the literature. Borderline Personality Disorder and Emotion Dysregulation, 2, 2. 10.1186/s40479-014-0024-3 26401305 PMC4579518

[jad12422-bib-0056] Poznyak, E. , Morosan, L. , Perroud, N. , Speranza, M. , Badoud, D. , & Debbané, M. (2019). Roles of age, gender, and psychological difficulties in adolescent mentalizing. Journal of Adolescence, 74, 120–129. 10.1016/j.adolescence.2019.06.007 31202040

[jad12422-bib-0057] Quek, J. , Bennett, C. , Melvin, G. A. , Saeedi, N. , Gordon, M. S. , & Newman, L. K. (2018). An investigation of the mentalization‐based model of borderline pathology in adolescents. Comprehensive Psychiatry, 84, 87–94. 10.1016/j.comppsych.2018.04.005 29727808

[jad12422-bib-0058] Rivnyák, A. , Pohárnok, M. , Péley, B. , & Láng, A. (2021). Identity diffusion as the organizing principle of borderline personality traits in adolescents—a non‐clinical study. Frontiers in Psychiatry, 12, 683288. 10.3389/fpsyt.2021.683288 34295274 PMC8289896

[jad12422-bib-0059] Ross, S. , & Heath, N. (2002). A study of the frequency of self‐mutilation in a community sample of adolescents. Journal of Youth and Adolescence, 31(1), 67–77. 10.1023/A:1014089117419

[jad12422-bib-0060] Rossouw, T. , Muir, O. , & Williams, L. L. (2020). Mentalization‐based treatment for adolescents: The framework for work with suicide and self‐injurious behaviors. In L. Williams , & O. Muir Eds, Adolescent suicide and self‐injury (pp. 125–136). Springer. 10.1007/978-3-030-42875-4_9

[jad12422-bib-0061] Rossouw, T. I. , & Fonagy, P. (2012). Mentalization‐based treatment for self‐harm in adolescents: A randomized controlled trial. Journal of the American Academy of Child & Adolescent Psychiatry, 51(12), 1304–1313.e3. 10.1016/j.jaac.2012.09.018 23200287

[jad12422-bib-0062] Sharp, C. , Pane, H. , Ha, C. , Venta, A. , Patel, A. B. , Sturek, J. , & Fonagy, P. (2011). Theory of mind and emotion regulation difficulties in adolescents with borderline traits. Journal of the American Academy of Child & Adolescent Psychiatry, 50(6), 563–573.e1. 10.1016/j.jaac.2011.01.017 21621140

[jad12422-bib-0063] Sharp, C. , Venta, A. , Vanwoerden, S. , Schramm, A. , Ha, C. , Newlin, E. , Reddy, R. , & Fonagy, P. (2016). First empirical evaluation of the link between attachment, social cognition, and borderline features in adolescents. Comprehensive Psychiatry, 64, 4–11. 10.1016/j.comppsych.2015.07.008 26298843

[jad12422-bib-0064] Sokol, Y. , & Eisenheim, E. (2016). The relationship between continuous identity disturbances, negative mood, and suicidal ideation. The Primary Care Companion for CNS Disorders, 18(1). 10.4088/PCC.15m01824 PMC4874761 27247841

[jad12422-bib-0065] Swannell, S. V. , Martin, G. E. , Page, A. , Hasking, P. , & St John, N. J. (2014). Prevalence of nonsuicidal self‐injury in nonclinical samples: Systematic review, meta‐analysis, and meta‐regression. Suicide & Life‐Threatening Behavior, 44(3), 273–303. 10.1111/sltb.12070 24422986

[jad12422-bib-0066] Turner, B. J. , Dixon‐Gordon, K. L. , Austin, S. B. , Rodriguez, M. A. , Zachary Rosenthal, M. , & Chapman, A. L. (2015). Non‐suicidal self‐injury with and without borderline personality disorder: Differences in self‐injury and diagnostic comorbidity. Psychiatry Research, 230(1), 28–35. 10.1016/j.psychres.2015.07.058 26315664

[jad12422-bib-0067] Verschueren, M. , Claes, L. , Bogaerts, A. , Palmeroni, N. , Gandhi, A. , Moons, P. , & Luyckx, K. (2018). Eating disorder symptomatology and identity formation in adolescence: A cross‐lagged longitudinal approach. Frontiers in Psychology, 9, 816. 10.3389/fpsyg.2018.00816 29915548 PMC5994691

[jad12422-bib-0068] Wolff, J. C. , Thompson, E. , Thomas, S. A. , Nesi, J. , Bettis, A. H. , Ransford, B. , Scopelliti, K. , Frazier, E. A. , & Liu, R. T. (2019). Emotion dysregulation and non‐suicidal self‐injury: A systematic review and meta‐analysis. European Psychiatry, 59, 25–36. 10.1016/j.eurpsy.2019.03.004 30986729 PMC6538442

[jad12422-bib-0069] Xiao, Q. , Song, X. , Huang, L. , Hou, D. , & Huang, X. (2022). Global prevalence and characteristics of non‐suicidal self‐injury between 2010 and 2021 among a non‐clinical sample of adolescents: A meta‐analysis. Frontiers in Psychiatry, 13, 912441. 10.3389/fpsyt.2022.912441 36032224 PMC9399519

[jad12422-bib-0070] Zeki, A. , & Çuhadaroğlu, F. Ç. (2012). *Developmental and psychopathological evaluation of non‐suicidal self‐injury among adolescents* [Unpublished specialization thesis]. Hacettepe University.

